# The Ten Commandments of Everyday Leadership

**DOI:** 10.1117/1.NPh.10.4.040101

**Published:** 2023-12-18

**Authors:** Anna Devor, Shadi Dayeh

**Affiliations:** a Boston University, College of Engineering, Department of Biomedical Engineering, Boston, Massachusetts, United States; b University of California in San Diego, Jacobs School of Engineering, Department of Electrical and Computer Engineering, La Jolla, California, United States

## Abstract

The editorial offers principles of everyday leadership, with hope for the new year.

**Figure:**
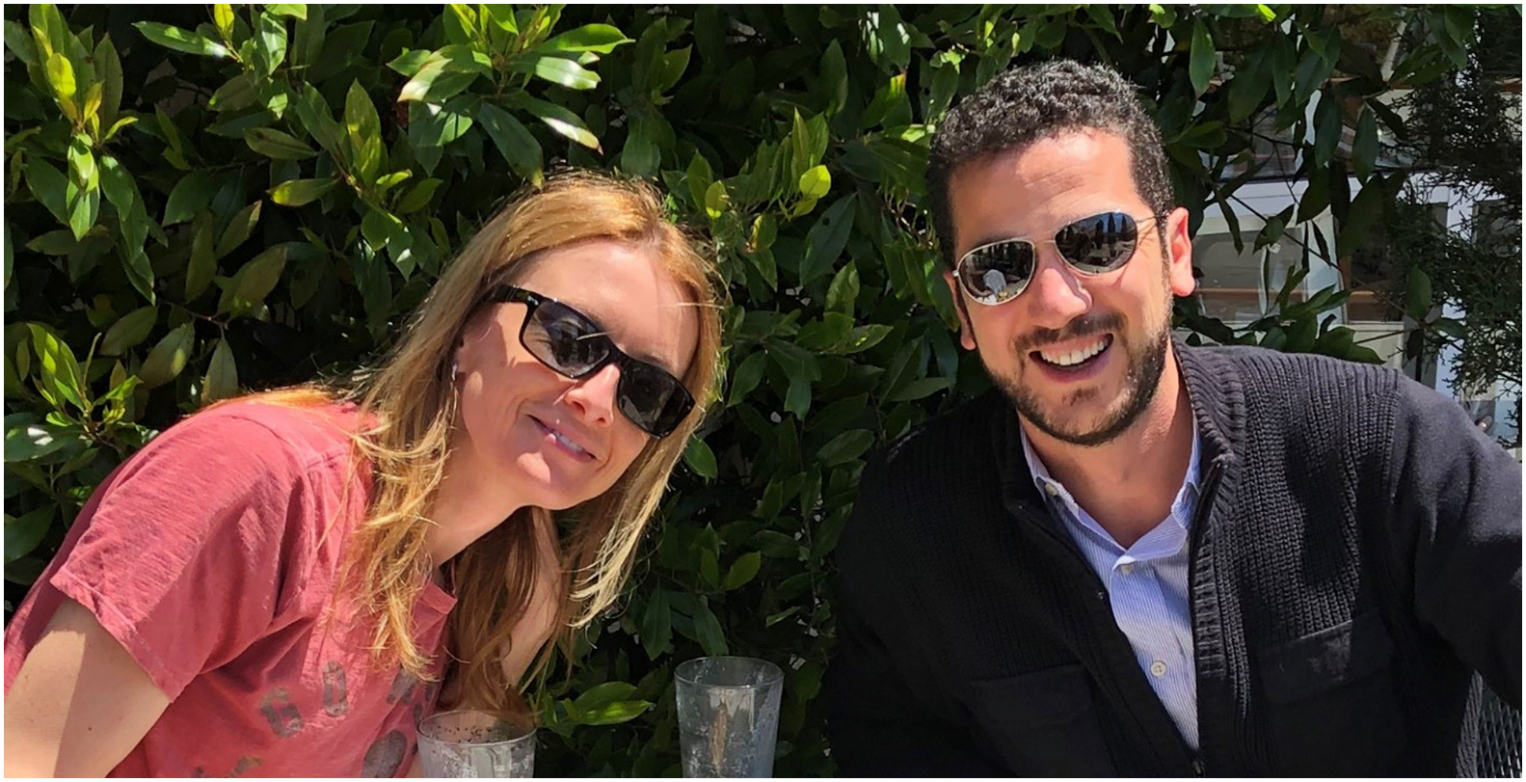
Anna Devor (left) is an Israeli-American neuroscientist, biomedical engineer, and Editor in Chief of *Neurophotonics*. Shadi Dayeh is a Lebanese-American materials and electrical engineer leading the Integrated Electronics and Biointerfaces Laboratory (IEBL) at University of California in San Diego.

It is this time of the year when many of us will have a chance to pause and catch a breath. It’s time to get together with family and friends, reflect on the year that passed, maybe make some New Year resolutions. This year, we feel that a break cannot come soon enough.

It has been an exhausting, tumultuous year with the war raging in Ukraine and Middle East and echoing across college campuses. So, maybe this is a good moment to stop in our daily tracks, turn off the light source on a vibration isolation table, and turn the attention to the light inside of us that is also susceptible to external vibrations but of a very different kind. What is this inner light? We call it hope. It is a universal and beautiful thing.

You, our Reader, possibly reside in a higher education institution. These institutions raise the next generation of our leaders. If you are faculty, you are a leader. If you are a trainee, you are on the way to leadership. So, let’s talk about that. Not the big questions of ideology, the world order, geopolitics, and religion – we believe that intellectual freedom is a hallmark of healthy democracies. Rather, let’s talk about principles of the everyday leadership that apply to our seemingly insignificant, minute-to-minute decisions and the way we interact with each other.

Of course, we (Anna and Shadi) are two imperfect individuals who really have no business lecturing others about anything outside of neuroimaging (Anna) and neurorecording (Shadi). But maybe this is exactly the point: we need these principles to guide ourselves, each of us trying to be a better person one day at a time. Here, we simply share them with you.

**Anna’s and Shadi’s Ten Commandments of Everyday Leadership**

1. Connect with your better angels and lead with love. Yes, we know that it’s hard. But positive messages inspire! Try it, and you’ll be surprised how much positive energy backscatters Right At Ya!
2. Having a difficult argument? Get a perspective. Some arguments change size with distance and change shape when they intersect planes of different cultures and personal identities. Yes, the world is an amazing place!
3. Before picking a side, consider that two things can be true at once. Need an example? A photon is both a particle and a wave!
4. Why would you even have a spectrometer if it wasn’t for the fact that the world isn’t black and white? Consider the full spectrum, an infinite number of frequencies and ways they combine!
5. We, people, are full of color, and each one takes their own, unique path colliding with obstacles, correcting trajectory, migrating through complex media. This is a diversity in its fullest! Avoid labels and see everyone for what they are.
6. Being on the opposite side of an argument does not make your opponent evil or unworthy. Ramón y Cajal and Camillo Golgi debated the contiguity of brain tissue. But what did Cajal use to make his most impactful observations? The Golgi stain!
7. Recognize when you do or do not have an informed opinion. Instant computing? Looking forward! Instant gratification? Doesn’t often happen in science. Instant opinion? Makes no sense whatsoever!
8. The space–time is a continuum, of course. But sometimes it’s helpful to focus on time. Need time to think and form your opinion? Take a step back. How much back? Well, this depends on the size of the problem. Having a really strenuous one? Back to the Big Bang you go!
9. It would only take 0.13 seconds for the light to circle the Earth. Yes, the world is a small place, we are all neighbors, and human feeling and aspirations are universal. Let’s be kind to each other!
10. Hope is a powerful force. In some sense, spirituality, inspiration, and creativity are all forms of hope. The world gets darker, and we become smaller, when we lose hope. Remember that light is our trade and don’t let it happen to you!

May the light be with you in 2024!

